# Bats, Emerging Diseases, and the Human Interface

**DOI:** 10.1371/journal.pntd.0000451

**Published:** 2009-07-28

**Authors:** Charles E. Rupprecht

**Affiliations:** Centers for Disease Control and Prevention, Atlanta, Georgia, United States of America; New York University School of Medicine, United States of America

## Background

The tragic death of a Dutch patient due to Duvenhage virus infection acquired after bat exposure in Kenya during 2007 emphasizes the potential dangers associated with ecotourism, underscores the role of Chiroptera as reservoirs of emerging infectious diseases, and highlights modern attempts to prevent and treat these zoonotic diseases.

The patient in this incident, a physician from The Netherlands, was a visitor to a game park in eastern Kenya, and had not been previously vaccinated against rabies. Often, many travelers abroad may not be well versed in local environmental conditions. Well before expected departure, travel medicine consultants should discuss both generic and country-specific risks with their clients. For maximum benefit, several biologics require administration a month in advance of departure, such as rabies pre-exposure immunization. Besides primary vaccination suggestions, and health insurance considerations for emergency care abroad or medical evacuation as needed, basic education is necessary concerning realistic public health concerns, especially in developing countries. Selective positive social behaviors should be promoted, especially as related to personal interactions with animals, both domestic and wild. Greater appreciation of animals from a distance is ideal, rather than any personal provocations. If animal bites or scratches do occur, immediate thorough washing of wounds with soap and clean water is valuable, followed by careful biomedical evaluation. While the latter was attempted in this particular Kenyan incident, the global implications of bat rabies were not appreciated.

Unlike the epidemiological situation in Europe and the developed world, rabid dogs remain responsible for the vast majority of human rabies cases in other parts of the world. For this reason, less attention is paid to infections acquired from other mammals, such as wildlife. With the exception of Antarctica, bat rabies occurs on all continents. The existence of lyssaviruses associated with infected Chiroptera in Africa has been documented for several decades [1]. Although surveillance is often limited or lacking, multiple studies to date suggest that bat rabies is much more widespread throughout Africa, and other continents, than is commonly communicated. In contrast to bites from mammalian carnivores, such as dogs, resulting injuries after even superficial direct contact with bats may appear rather trivial by comparison, as illustrated in this patient, with only superficial wounds noticed on her nose. In another typical example from Texas in 2006, a teenager died from rabies 1 month after a report of a bat that had awakened him, after landing upon his face [2]. Clearly, based upon both experimental and epidemiological criteria, any probable likelihood for bona fide transdermal or mucosal exposures to a bat is a reasonable consideration for rabies prophylaxis, regardless of geographical locality. Confusion in the use of specific viral taxonomy related to the presence or absence of “rabies virus” per se, versus other “lyssaviruses”, or bio-political considerations over so-called “rabies-free” areas, are largely irrelevant to the health provider and traveler alike, and pale in the face of obvious public health concerns when ignored or misunderstood, in the wake of volant reservoirs. To simplify: rabies is an acute progressive encephalitis; the clear majority of cases occur after animal bite; lyssaviruses are the neurotropic etiological agents that cause rabies; rabies virus is only one of at least 11 types of lyssavirus; rabies is a preventable infectious disease; any suggestions of “rabies-like diseases” are obvious misnomers that only obfuscate health communications.

## Findings

The reported incubation period in the Dutch patient of approximately 3 weeks after bat exposure to her face falls within the expected 1–3 month estimate for rabies. In the light of a suggestive history and compatible clinical signs, human antemortem diagnostics attempt to define specific lyssavirus antigens, antibodies, or amplicons from clinical material, including skin biopsy, serum, cerebrospinal fluid, and saliva, but rarely follow a discrete predictable course given the diversity of lyssavirus variant, route, dose, and patient parameters. Host infection and immunity related to lyssaviruses are complex, multigenic events [3]. After exposure, viral RNA may persist locally in tissues for days to weeks [4]. Apparent abortion of productive infections and induction of virus-neutralizing antibodies in seemingly healthy animal populations have been detected historically in taxa as diverse as bats and mongoose [5],[6]. Such observations have even been extended to a few human populations at risk, such as those involved with animal trapping [7],[8]. The innate and adaptive mechanisms against rabies are poorly understood, which include outcomes of protective immunity sans overt illness, clinical recovery, or death [9]. Animals have recovered after experimental rabies, usually with frank neurological sequellae [10]. Spontaneous recoveries after overt illness, with involvement of the central nervous system, have not been well documented in human rabies infections. However, considering the genetic and antigenic diversity of lyssaviruses, coupled with the plasticity of the mammalian immune response, recovery from such a lyssavirus-induced encephalitis is conceptually appealing, albeit rare in actuality. The fact that as little as a single base change in the genome equates with the difference between a pathogenic virus and a highly attenuated agent strongly suggests that a continuum exists between virulence and immunity resulting from more “temperate” lyssaviruses, at least in experimental settings [11],[12].

The patient did begin rabies prophylaxis, but only after the advent of encephalitis. Rabies immunization is highly effective, if administered to those at risk before exposure, or promptly and properly after viral exposure. Typical human postexposure prophylaxis entails immediate wound care, the infiltration of rabies immune globulin in and around the bite, and the parenteral inoculation of cell culture vaccine on days 0, 3, 7, 14, and 28 (although the absolute number of doses, routes, and schedules may vary globally). Postexposure prophylaxis should begin as soon as possible after exposure, and should not be withheld even with the passage of time after a bite, as virus may reside locally for several days after exposure, even though the likely effectiveness of intervention is expected to decline as virus accesses the nervous system. Prophylaxis is not appropriate in rabies patients after the advent of clinical signs. Such utilization has not been shown to be effective in either actual clinical settings or in experimental applications with laboratory animals. As such, administration of prophylaxis concomitant with illness onset offers false hope to families without substantiation and in the context of a developing country would waste expensive valuable biologics. Moreover, such vaccinations can interfere with diagnostic testing on patient sera, as well as confound interpretations if experimental therapeutics are elected. Critically, from the standpoint of acute outcome, application of such inactivated commercial biologics may skew host immunity from a more appropriate response and actually threaten patient health, within the realms of the “early death” phenomenon [13].

The dual administration of human rabies immune globulin and vaccine, and institution of experimental treatment, in this critically ill patient was a desperate attempt to intervene against an otherwise fatal disease. Human rabies treatment, as practiced favorably upon an unvaccinated 15-year-old girl bitten on her finger by a bat in Wisconsin during 2004, is challenging, expensive, and no simple panacea. While not successful in this Dutch case or in several other patients as can be gleaned from the Wisconsin protocol patient registry, likely differences in etiology, exposure route, infectious dose, host factors, timing of intervention, nosocomial issues, and other complex variables prevent a simple comparison to the so-called original Milwaukee protocol. At a minimum, proper palliative comfort care needs to be offered to all rabies patients. The value of any experimental intervention for this disease remains to be proven ultimately based upon further scientific insights, the tincture of time, and a gradual accumulation of successes from those that dare to risk non-conventional approaches and attempt to overcome the near impossible statistics associated with this malady [14],[15].

## Implications

The failure of a successful outcome with this particular patient does showcase one fatal conundrum in the state of the art of research in rabies therapeutics. Animal models have played a large role in the experimental development of biologics against rabies, especially in vaccine production. However, to design a successful intervention against clinical rabies, new paradigms are needed. A historical focus upon fixed, laboratory rabies viruses, intracerebral inoculation, and the utilization of laboratory mice alone have provided some insights into basic pathogenesis, but are quite limited from the standpoint of street virus heterogeneity, more natural routes of exposure, and logistical limitations of medical care in small-bodied mammalian subjects. The utilization of more appropriate species would allow greater use of intensive, synergistic clinical intervention in rabid subjects at different stages of disease onset, as would be experienced in a sophisticated intensive care setting, coupled with promising compounds derived from a more rational, targeted approach in anti-viral design [16],[17].

## Future Directions

The lessons learned from this fatal human case after exposure to a rabid African bat are multiple, including the following: improved support for a multidisciplinary approach towards relevant health communications on the existence of emerging pathogens abroad, especially as related to bats and the prevention of such deleterious outcomes; greater basic research on pathogenic mechanisms associated with such agents, particularly as regards an extension to proximate biomedical interventions, once clinical signs manifest; and an integrated applied outreach on the ecology of zoonotic and vector-borne infectious diseases for improved long-term prevention and control strategies, within an encompassing “one health” philosophy.
